# ﻿*Begoniaparvibracteata*, a new species in ﻿*Begonia* sect. ﻿*Platycentrum* (Begoniaceae) from Guangxi of China, based on morphological and molecular evidence

**DOI:** 10.3897/phytokeys.214.90004

**Published:** 2022-11-22

**Authors:** Xin-Xin Feng, Xiao-Feng Huang, Yu-Ni Huang, Zhi-Xian Liu, Ren-Kun Li, Jin-Ye Zhou, Wei Guo, Xiao-Yan Chen, Dai-Ke Tian

**Affiliations:** 1 Dongguan Botanical Garden, Dongguan 523086, China Dongguan Botanical Garden Dongguan China; 2 Enshi Dongsheng Plant Development Co. Ltd., Enshi 445000, China Enshi Dongsheng Plant Development Co. Ltd. Enshi China; 3 Flower Research Institute, Guangxi Academy of Agricultural Sciences, Nanning 530007, China Flower Research Institute, Guangxi Academy of Agricultural Sciences Nanning China; 4 Department of Horticulture and Landscape Architecture, Zhongkai University of Agriculture and Engineering, Guangzhou 510225, China Zhongkai University of Agriculture and Engineering Guangzhou China; 5 Guangdong Joco Eco-Environment Co., Ltd, Dongguan 523012, Guangdong, China Guangdong Joco Eco-Environment Co., Ltd Dongguan China; 6 Shanghai Chenshan Plant Science Research Center, Chinese Academy of Sciences, 3888 Chenhua Road, Songjiang, Shanghai 201602, China Shanghai Chenshan Plant Science Research Center, Chinese Academy of Sciences Shanghai China; 7 Shanghai Key Laboratory for Plant Functional Genomics and Resources, Shanghai Chenshan Botanical Garden, 3888 Chenhua Road, Songjiang, Shanghai 201602, China Shanghai Key Laboratory for Plant Functional Genomics and Resources, Shanghai Chenshan Botanical Garden Shanghai China

**Keywords:** ITS, morphology, new taxon, southern China, taxonomy

## Abstract

The previously reported begonias in a limestone forest of Guangxi mainly belong to Begoniasect.Coelocentrum Irmscher. In this article, we described and illustrated a new species in sect. Platycentrum (Klotzsch) A.DC., *Begoniaparvibracteata* X.X.Feng, R.K.Li & Z.X.Liu, which was discovered in a karst forest of south-western Guangxi. The begonia shows high morphological similarity to *B.subhowii* S.H. Huang and *B.psilophylla* Irmscher, but differs from the latter two in its narrower oblique-ovate asymmetric leaf blade, 4 (occasionally 6) tepals of pistillate flower and smaller membranous inflorescence bracts. Molecular phylogenetic analysis, based on ITS sequence data, supports the new species as monophyletic and distinct from *B.subhowii* and *B.psilophylla*. Considering its narrow distribution and the disturbance of human activities, the conservation status of new taxon is evaluated as “Vulnerable” (VU B1, B2 ab (i, iv, v), D2) according to the IUCN Red List Categories and Criteria.

## ﻿Introduction

According to the latest report, China’s wild begonias have already increased to 239 species (iBegonia 2021). The total number of species could reach 300 in the coming years, meeting the prediction of Tian et al. (2018). Consisting of 122 species, Sect. Platycentrum (Klotzsch) A.DC. represents the largest section of *Begonia* in China, with 66 species mainly distributed in Yunnan. Eighty-seven species of *Begonia* have so far been found in Guangxi (iBegonia 2021), most of which (60 species) are distributed in the karst forest and belong to B.sect.Coelocentrum Irmscher.

In Guangxi, only 11 species belong to B.sect.Platycentrum (Klotzsch) A.DC., and six of which are widespread in south and southeast China: *B.circumlobata* Hance, *B.edulis* Lévl., *B.handelii* Irmsch., *B.hemsleyana* Hook.f., *B.longiciliata* C.Y.Wu and *B.longifolia* Blume. Only five species of B.sect.Platycentrum are endemic to Guangxi, including *B.tsoongii* C.Y.Wu (Wu and Ku 1995), *B.longanensis* C.Y.Wu (Wu and Ku 1997), *B.auror*a C.I Peng, Yan Liu & W.B. Xu (Liu et al. 2020), *B.scorpiuroloba* D.K.Tian & Q.Tian (Tian et al. 2021) and *B.pseudoedulis* D.K.Tian, X.X.Feng & R.K.Li (Feng et al. 2021).

An unknown *Begonia* taxon with reproductive organs was collected from Guangxi during our field survey and plant collection in May 2020 and October 2021, respectively. The begonia definitely belongs to B.sect.Platycentrum, but differs markedly from the reported 11 begonias of the same section in Guangxi. After further detailed morphological observation, morphological comparison with similar species and molecular analysis, it is confirmed as a new species in Begoniasect.Platycentrum.

## ﻿Materials and methods

### ﻿Taxonomic observation

Morphological characters were observed and measured from fresh samples in the field. Morphological comparisons with similar taxa were undertaken by consulting the literature, examining herbarium (IBK and IBSC) specimens and observing living collections cultivated in the nursery of Enshi Dongsheng Plant Development Co. Ltd. The specimens were deposited at the South China Botanical Garden (**IBSC**), CAS and Chenshan Herbarium (CSH) of Shanghai Chenshan Botanical Garden.

### ﻿DNA sequencing and molecular analysis

The fresh leaves of the putative new species and the morphologically similar allied species, *B.subhowii* and *B.psilophylla*, were collected in the field and the nursery, respectively. Total DNA was extracted from nine individuals of the three species (*B.parvibracteata*, *B.subhowii* and *B.psilophylla*) with the CTAB method (Doyle and Doyle 1987). The internal transcribed spacers 1 and 2 (ITS) and the 5.8S gene were amplified using the primers 17SE and 26SE (Forrest and Hollingsworth 2003). PCR amplification and Sanger sequencing were performed according to Fan et al. (2014), with an annealing temperature of 53 °C. The ITS sequences of the three species were uploaded to GenBank (http://www.ncbi.nlm.nih.gov/) with the accession numbers OL892048, OL892049 and OL892050 for *B.parvibracteata* sp. nov., OL871361, OL871362 and OL871363 for *B.subhowii* and OL851701, OL851702 and OL851703 for *B.psilophylla* (Table 1).

**Table 1.** *Begonia* species and populations included in the phylogenetic analysis (Sectional placement follows Moonlight et al. 2018).

Taxon	Origin	GenBank accession no.	Section	Collector, voucher (Herbarium)
*Begoniaacetosella* Craib.	Mengla, Yunnan, China	MW690106	*Platycentrum*	Wang, W.G., WWG005 (HITBC)
*Begoniabiflora* Ku	Malipo, Yunnan, China	JF975965	*Coelocentrum*	Shui, Y.M. et al. 20484 (KUN)
*Begoniachingii* Irmsch.	Napo, Guangxi, China	KP710820	*Reichenheimia*	Tian, D.K., Li, C. TDK785 (CSH)
*Begoniacircumlobata* Hance	Xinyi, Guangdong, China	KP710815	*Platycentrum*	Tian, D.K., Li, X.P. TDK866 (CSH)
*Begoniacucurbitifolia* C. Y. Wu	Yunnan,China	JF975969	*Platycentrum*	Y,M,Shui et al.GBOWS1284 (KUN)
	–	JF975968		–
*Begoniaedulis* Lévl.	Bama, Guangxi, China	KP710813	*Platycentrum*	Tian, D.K., Li, C. TDK757 (CSH)
*Begoniagrandis* Dry.	Yongshun, Huhan, China	KP710828	*Diploclinium*	Li, X.P. Li, X.J. LXJ022 (CSH)
*Begoniahandelii* Irmsch.	Fengshan, Guangxi, China	KP710818	*Platycentrum*	Tian, D.K., Li, C. TDK763 (CSH)
*Begoniahatacoa* Buch.-Ham. ex D. Don	–	AF485111	*Platycentrum*	–
*Begoniahemsleyana* Hook. f.	–	KP710806	*Platycentrum*	–
*Begoniahenryi* Hemsl.	Leshan, Sichuan, China	KP710822	*Reichenheimia*	Tian, D.K., Tian, L.Z. TDK2249 (CSH)
*Begoniahuangii* Y. M. Shui & W. H. Chen	Gejiu, Yunnan, China	JF976001	*Coelocentrum*	Shui, Y.M. et al. 40782 (KUN)
*Begoniajinyunensis* C. I Peng, B. Ding & Q. Wang	Jinyunshan, Chongqing, China	MZ145345	*Platycentrum*	–
*Begonialabordei* Lévl.	Yunnan, China	KF636452	*Diploclinium*	Peng 20520 (HAST)
*Begonialongifolia* Blume	Mengla, Yunnan, China	MW690102	*Platycentrum*	Wang, W.G., WWG001 (HITBC)
*Begonialongistyla* Y. M. Shui & W. H. Chen	Hekou, Yunnan, China	JF976018	*Coelocentrum*	Shui, Y.M. et al. 40778 (KUN)
*Begoniamegalophyllaria* C. Y. Wu	Yunnan, China	JF976026	*Platycentrum*	Y,M,Shui et al.D-33(KUN)
*Begoniamultangula* Blume	Jawa, Lesser Sunda Is.	MN453434	*Platycentrum*	–
*Begoniaornithophylla* Irmsch.	Guangxi, China	JF976032	*Coelocentrum*	Y,M,Shui et al. B2005-061(KUN)
*Begoniaparvibracteata* X.X.Feng, R.K.Li & Z.X.Liu	Longzhou, Guangxi, China	OL892048	*Platycentrum*	Xin Xin Feng, et al. 835307 (IBSC)
		OL892049		
		OL892050		
*Begoniapedatifida* Lévl.	Tianlin, Guangxi, China	KP710810	*Platycentrum*	Tian, D.K., Li, C. TDK774 (CSH)
*Begoniapsilophylla* Irmsch.	Hekou, Yunnan, China	OL851701	*Platycentrum*	–
		OL851702		–
		OL851703		–
*Begoniapulchrifolia* D.K.Tian & C.H.Li	Meinvfeng, Leshan, Sichuan, China	KP710811	*Platycentrum*	Tian, D.K., et al. TDK2243 (CSH)
*Begoniascorpiuroloba* D.K.Tian & Q.Tian	Fanchenggang, Guangxi, China	MZ145351	*Platycentrum*	Tian, D.K., et al.TDK2269(CSH)
*Begoniasocotrana* Hook.f.	Socotra	AF469121	*Peltaugustia*	–
*Begoniasubhowii* S. H. Huang	Malipo, Yunnan, China	OL871361	*Platycentrum*	–
		OL871362		–
		OL871363		–

We chose 17 species from sect. Platycentrum and 8 species from other sections of *Begonia* native to mainland China to place the new species in a phylogenetic context (Table 1). *Begoniasocotrana* Hook.f in sect. Peltaugustia (Warb.) Barkley from Socotra (for the coast of Africa) was selected as an out-group in the phylogenetic analysis (Moonlight et al. 2018). Except for the nine individuals of the putative new species and its two allied species, the additional ITS sequences for 23 *Begonia* species were downloaded from the NR database of NCBI. All these sequences were aligned using BioEdit v.7.2.5 (Hall 1999) and a phylogenetic analysis using Bayesian Inference (BI) was undertaken in MrBayes v.3.1.2 (Ronquist and Huelsenbeck 2003). The GTR+G model was chosen as the optimal model of nucleotide substitution according to the Akaike Information Criterion (AIC; Burnham and Anderson 2002) as implemented in MrModeltest 2.3 (Nylander 2004). The Markov chains were run for 1,000,000 generations and sampled at each 100 generations, with the first 25% discarded as burn-in.

## ﻿Taxonomy

### ﻿ Begonia parvibracteata

Taxon classificationPlantaeCucurbitalesBegoniaceae

X.X.Feng, R.K.Li & Z.X.Liu sp. nov.

64EF92FB-29EF-5C54-95F8-45862A64093D

urn:lsid:ipni.org:names:77308553-1

**Figure 1.**
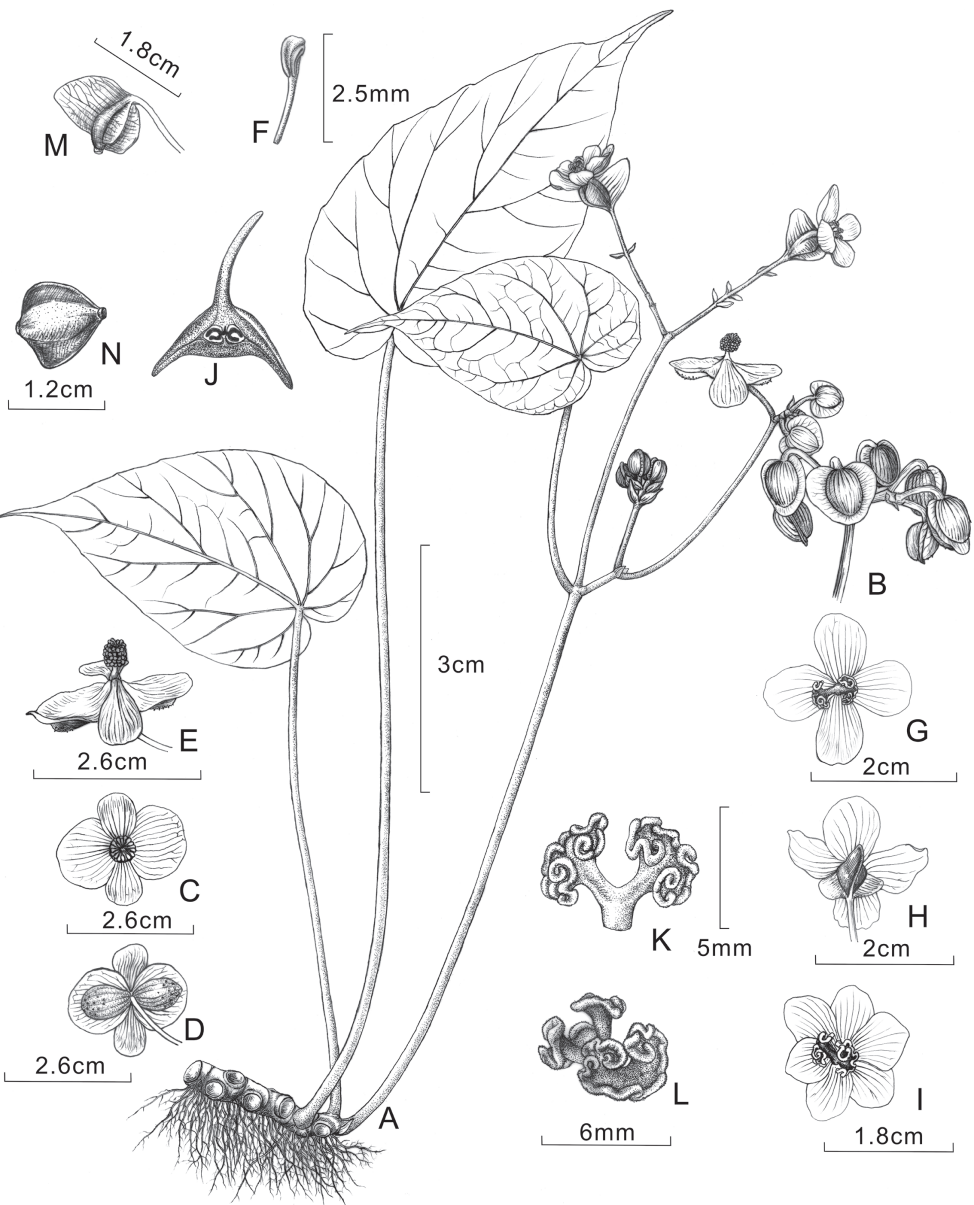
*Begoniaparvibracteata* drawn by Yunxiao Liu **A** хabit **B** staminate inflorescence **C, D** front and back views of staminate flower **E** lateral view of staminate flower **F** stamen **G, H** front and back views of pistillate flower with 4 tepals **I** front view of pistillate flower with 6 tepals **J** cross section of ovary **K** styles and stigmas **L** stigmas **M** abaxial view of capsule showing wings **N** lateral view of capsule showing two shorter wings.

**Figure 2.**
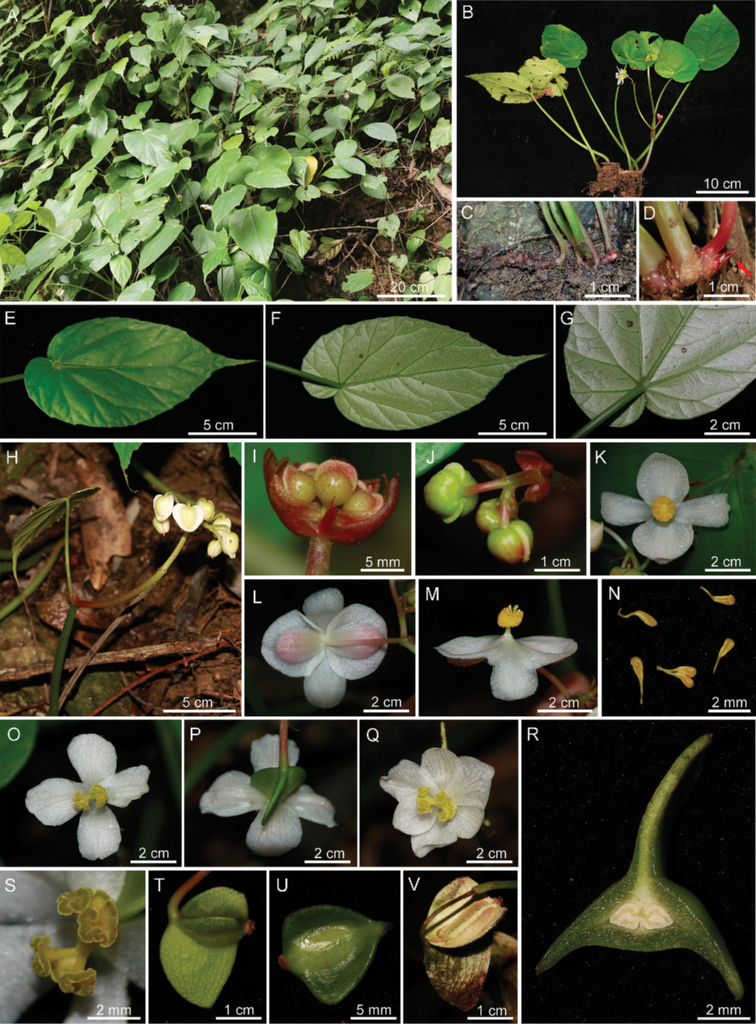
Habitat and morphology of *Begoniaparvibracteata***A** habitat **B** flowering plant **C** creeping rhizome **D** shoot top with stipule **E** leaf blade (adaxial) **F, G** leaf blade (abaxial) **H** erect stem with inflorescence **I, J** young inflorescence and bract; **K, L** front and back views of staminate flower with 4 tepals **M** lateral view of staminate flower **N** stamens **O, P** front and back views of pistillate flower with 4 tepals **Q** front view of pistillate flower with 6 tepals **R** cross section of ovary **S** stigmas **T, U** immature capsule **V** dried mature capsule (Photos by Z.X. Liu).

Figs 1 , 2 Chinese name: 小苞秋海棠

#### Type.

**Figure 3.**
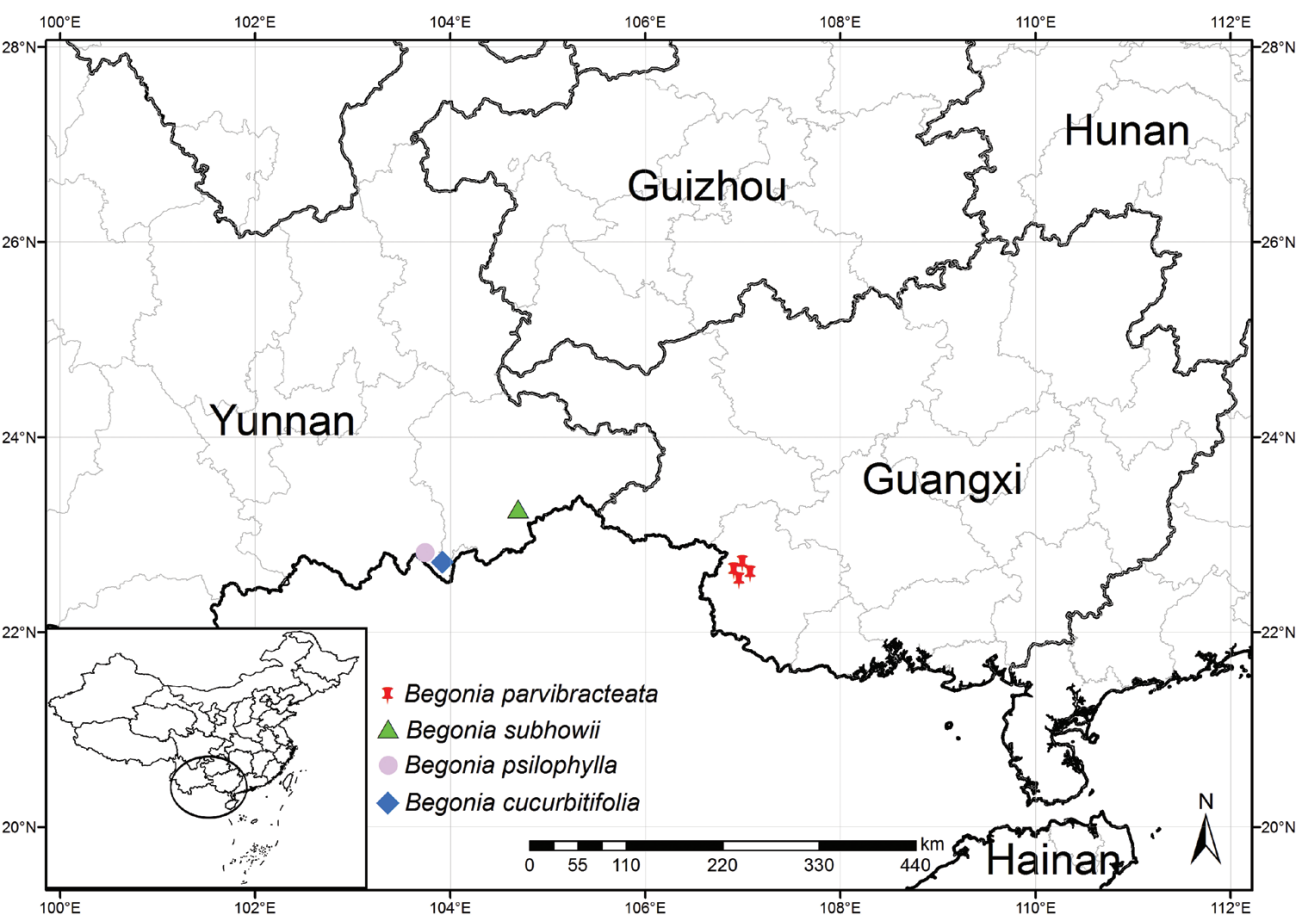
Distribution of *B.parvibracteata*, *B.subhowii*, *B.psilophylla* and *B.cucurbitifolia*.

**China** Guangxi, Longzhou County (龙州县), Zhubu Town (逐卜乡), Bannong Village (板弄屯), Yinghuagu Tourist Resort, 22°33'51"N, 106°57'03"E, (Fig. 3), 263 m alt., in shaded environment of limestone forest, October, 2021, *Xin-Xin Feng*, *Ren-Kun Li* & *Zhi-Xian Liu* (holotype: 835307, IBSC!; isotype: SYS!). Longzhou County (龙州县), Zhubu Town (逐卜乡), Pona Village (坡那屯), Nonggang National Nature Reserve, 22°39'03"N, 106°57'18"E, 190 m alt., on rock under limestone forest, 18 May 2020, *Dai-Ke Tian* & *Jinye Zhou* TDK4119 (CSH!).

#### Diagnosis.

*Begoniaparvibracteata* morphologically resembles *B.subhowii* and *B.psilophylla* in rhizome and leaf characters. However, it has narrowly oblique-ovate asymmetric leaf blades, 4 (rarely 6) tepals in pistillate flowers and small (6–8 × 3–5 mm) bracts in inflorescences. These characters differ from the widely ovate leaf blades, 5 (rarely 6) tepals of pistillate flowers, and distinctly large bracts in inflorescences of *B.subhowii*. *B.parvibracteata* is dissimilar to *B.psilophylla* in its 4 (rarely 6) tepals in pistillate flowers and asymmetric, narrowly oblique-ovate leaf blade.

*Begoniaparvibracteata* forms a monophyletic group clustered with *B.cucurbitifolia* in the phylogenic tree, but the latter has nearly symmetric, 3–4 lobed leaf blade, 5-tepaled pistillate flower, definitely differing from the new species.

#### Description.

Perennial evergreen herb, monoecious, 15–30 cm tall. ***Rhizome*** creeping, ca. 8.5–12 cm long and 6–10 mm thick, internode short or not obvious; erect stem only seen at anthesis, ca. 7.5–10 cm long, internodes 1–2 (3), green to reddish-green, glabrous. ***Stipules*** brownish-red, translucent, ovate-triangular, ca. 8–10 × 4–5 mm, glabrous. ***Leaves*** 3–6 basal and 2–3 aerial, petiole yellowish-green, 18–22 cm long, glabrous; blade asymmetric, ovate to narrowly ovate, 14–16 × 7.5–9 cm; apex acuminate to caudate, base oblique-cordate; leaf margin usually almost entire or occasionally crenate; venation palmate with 6–8 primary veins, adaxially slightly concave, abaxially convex; leaf blade fleshy, abaxially pale-green, glabrous; adaxially green, glabrous. ***Inflorescences*** arising from erect stem; dichasial cymes branching one to two times, peduncle 10–12 cm long, glabrous; flowers unisexual, 3–7 flowers per inflorescences; bracts membranous, triangular to widely ovate, brownish-red, 6–8 × 3–5 mm, glabrous. ***Staminate flower***: pedicels pale green, ca. 2–2.3 cm long, glabrous; tepals 4, outer 2 tepals ovate to circular, 13–21 × 12–20 mm, adaxially concave, pinkish-white, abaxially convex, pinkish-white, red hispid; inner 2 obovate, 10–18 × 8–12 mm, margin irregularly entire, white, glabrous; androecium cylindrical, ca. 6.6–7.5 mm across; stamens numerous, ca. 2–2.5 mm long, anthers yellow, clavate, base cuneate, ca. 1.5 mm long. ***Pistillate flower***: pedicels pale green, ca. 2–2.6 cm long, glabrous; tepals 4, occasionally 6, white, irregularly ovate, obovate or cuneate, sub-equal, 18–20 × 10–15 mm, glabrous; ovary yellowish-green, trigonous-ellipsoid, 11–12 × 5–6 mm (wings excluded), glabrous; 2-loculed, placentae axillary, placentae bifid per locule; styles 2, fused at base, yellow, ca. 5–6 mm long, apically Y-shaped, stigma U-shaped, spirally twisted. ***Capsules*** nodding, trigonous-ellipsoid, ca. 11–13 × 5–6.5 mm (wings excluded), yellowish-green, glabrous, unequally 3-winged, abaxial wing triangular to ligulate, ca. 10–13 mm long; lateral wings lunate, 3–6 mm long.

#### Distribution and habitat.

Currently known from four localities in Longzhou County and Daxin Countym Guangxi, China. It usually grows on rocks or rock cracks in limestone under forest.

#### Phenology.

Flowering September-October, fruiting October-December.

#### Etymology.

The specific epithet “*parvibracteata*” refers to the short small bracts of the new species. The Chinese name is given as “小苞秋海棠” (Begonia with small inflorescence bracts).

#### Conservation status.

There are three populations with approximately 1000 individuals found in Longzhou County. Another one population with approximately 500 individuals is distributed in the Encheng National Nature Reserve, Daxin County. Some plants of this begonia are over-collected and sold in the local medicinal herb market of Longzhou County. According to the IUCN Red List Categories and Criteria (IUCN 2022), *B.parvibracteata* should be assessed as “Vulnerable (VU B1, B2 ab (i, iv, v), D2)” due to its narrow distribution and the disturbance by human activities.

##### ﻿Molecular analysis

**Figure 4.**
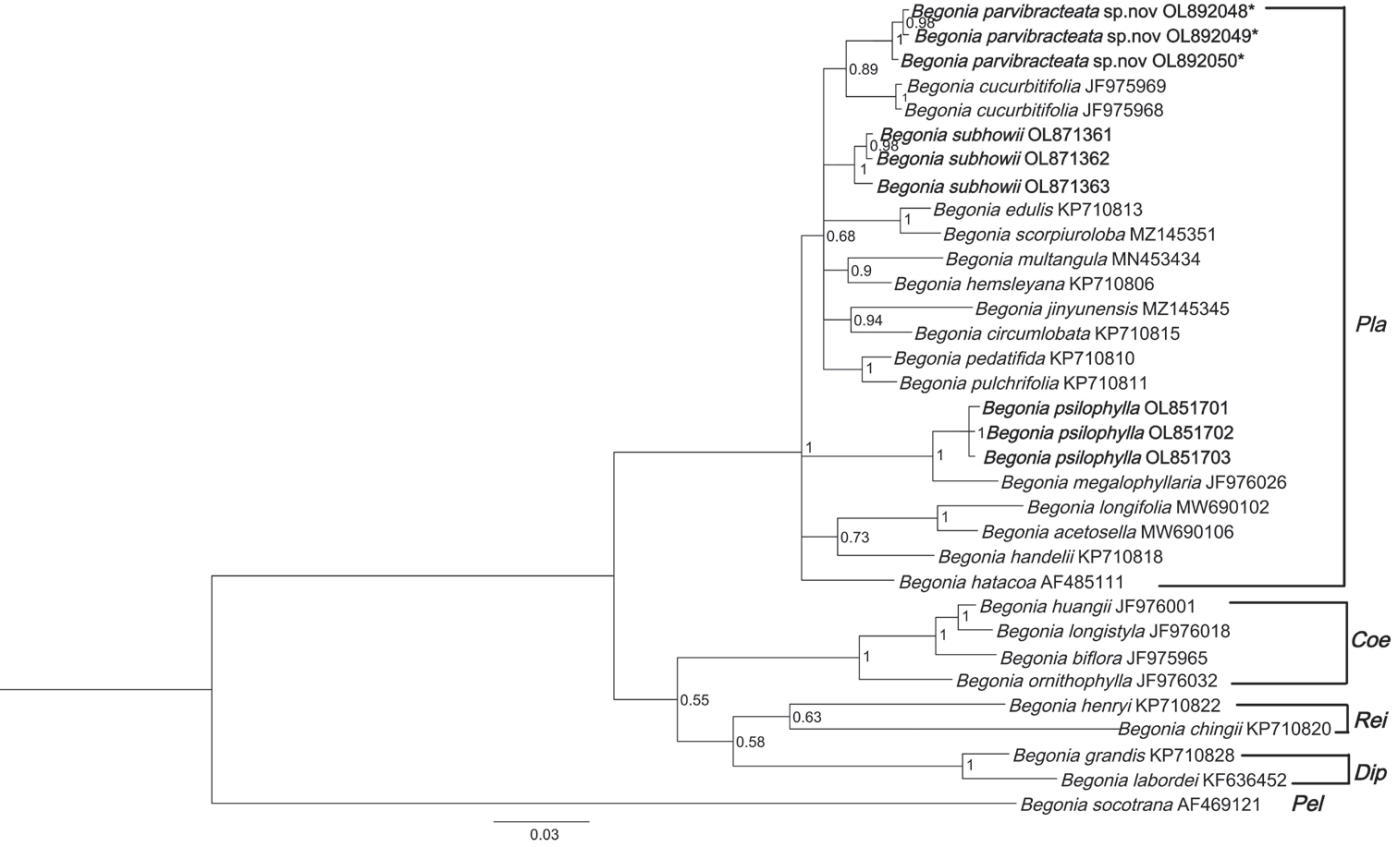
Bayesian Inference of the phylogenetic position of the newly-described *B.parvibracteata* within sect. Platycentrum, based on nuclear ITS sequences.

The aligned matrix of the ITS sequence data was 727 bp long. The result of Bayesian Inference analysis is shown in Fig. 4. Begoniasect.Platycentrum appears monophyletic with a high Bayesian posterior probability (bpp = 1) (Fig. 4). The three samples of the putative new species form a monophyletic group clustered with *B.cucurbitifolia* (bpp = 0.89). *Begoniasubhowii* with the highest morphological similarity to *B.parvibracteata* formed another subclade.

The nodes with bpp < 0.50 have been collapsed. Sectional placement of taxa is indicated by the following abbreviations: Coe (Coelocentrum), Dip (Diploclinium), Pla (Platycentrum), Rei (Reichenheimia) and Pel (Peltaugustia). The numbers after the species names indicate different populations. The samples of the new species are marked with stars.

## ﻿Discussion

The new begonia with 2-loculed ovary, axillary placentation and bifid placentae can be clearly assigned to B.sect.Platycentrum (Gu et al. 2007). Within this largest section for *Begonia* in China, *B.parvibracteata* shows high morphological resemblance to *B.subhowii* and *B.psilophylla*, both of which are distributed in south-eastern Yunnan (Shui and Huang 1999; Shui and Chen 2018), including creeping rhizome, glabrous plants with both basal and cauline leaves at anthesis, usually almost entire leaf margin, palmate venation, fleshy green leaf blade and 4 tepals of staminate flower (Table 2).

**Table 2.** Morphological comparison of *B.parvibracteata* and relevant taxa.

Character	*B.subhowii*	*B.psilophylla*	*B.cucurbitifolia*	*B.parvibracteata*
**Leaf blade shape**	asymmetric, widely ovate	nearly symmetric, widely ovate to cordate	nearly symmetric, orbicular, 3–4 lobed	asymmetric, narrowly oblique-ovate
**Leaf blade base**	asymmetric, oblique-cordate	nearly symmetric, obtuse	nearly symmetric, cordate	asymmetric, oblique-cordate
**Leaf margin**	irregularly serrulate	minutely serrulate	minutely serrulate	usually almost entire or occasionally crenate
**Inflorescence bracts**	ovate-oblong, 50 × 30 mm, glabrous, apex acuminate	elliptic to ovate-oblong, 7–9 × 4–5 mm, glabrous	subglabrous, caduceus	triangular, 6–8 × 3–5 mm, glabrous
**Tepal number of pistillate flowers**	usually 5, rarely 6	usually 5, rarely 6	5	usually 4, rarely 6
**Phenology (flowering; fruiting)**	April-May; May-July	February-March; March-May	July-August; August-October	September-October; October-December
**Habitat**	700–1500 m alt., limestone, Yunnan; Vietnam	100–700 m alt., limestone, Yunnan	430 m alt., limestone, Yunnan	263 m alt., limestone, Guangxi

**Figure 5.**
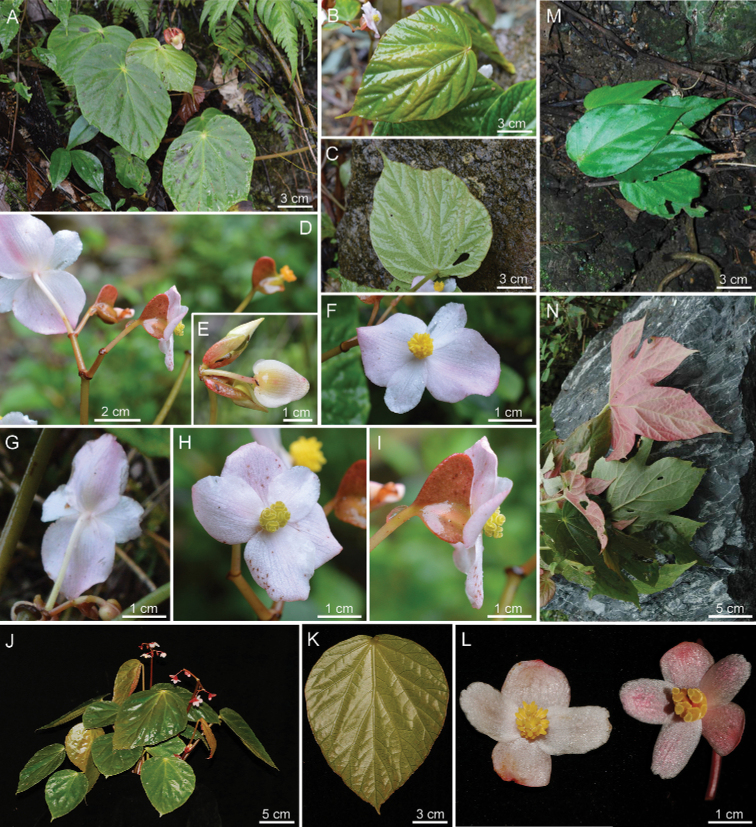
*B.subhowii***A–I***B.psilophylla***J–L***B.cucurbitifolia***M, N** showing similar features comparable to *B.parvibracteata***A** habitat **B** young leaf blade (adaxial) **C** leaf blade (abaxial) **D** inflorescence **E** bracts **F, G** front and back views of staminate flower **H** front view of pistillate flower with 5 tepals **I** lateral view of pistillate flower **J** flowering plant **K** symmetric leaf blade **L** front view of flower **M** leaf blade of juvenile plant **N** mature leaf blade (Photos **A–I** by R.K. Li **J–L** by Z.X. Liu **M, N** by D.K. Tian).

In *B.subhowii*, there are 5 (occasionally 6) tepals in the pistillate flower and the bracts of inflorescence is distinctly long and large (5 × 3 cm) (Fig. 5). These characters differ from 4 (occasionally 6) tepals and triangular to widely ovate (6–8 × 3–5 mm) bracts in *B.parvibracteata*. In addition, the leaf blade is wider ovate in *B.subhowii*, compared with the narrower oblique-ovate shape of *B.parvibracteata*.

In *B.psilophylla*, the leaf blade is nearly symmetric, widely ovate with symmetric cordate base, in contrast with the narrower ovate leaf blade and oblique-cordate base of the new species. Furthermore, there are 5 (occasionally 6) tepals in the pistillate flower of *B.psilophylla*, being different from the 4 (occasionally 6) tepals of *B.parvibracteata*.

According to the phylogenetic tree, based on ITS sequences, the closest relative of *B.parvibracteata* is *B.cucurbitifolia* which is also distributed in south-eastern Yunnan. However, *B.cucurbitifolia* is remarkably distinct from *B.parvibracteata* in morphology, including nearly symmetric, 3–4 lobed leaf blade and 5-tepaled pistillate flower.

For the flowering time, *B.parvibracteata* blooms later compared with its three allied species mentioned above.

## Supplementary Material

XML Treatment for Begonia parvibracteata

## References

[B1] Burnham KP Anderson DR (2002) Model Selection and Multimodel Inference: a practical information-theoretic approach. Springer, New York, USA, 488 pp.

[B2] Doyle JJ Doyle JL (1987) A rapid DNA isolation procedure for small quantities of fresh leaf tissue. Phytochemical Bulletin 19: 11–15.

[B3] Fan Q Chen SF Li MW Guo W Jing HJ Wu W Zhou RC Liao WB (2014) Molecular evidence for natural hybridization between wild loquat (*Eriobotryajaponica*) and its relative *E.prinoides*. BMC Plant Biology 14(1): 275. 10.1186/s12870-014-0275-6 25300306 PMC4196008

[B4] Feng XX Xiao Y Liu ZX Li RK Wei D Tian DK (2021) *Begoniapseudoedulis*, a new species in Begoniasect.Platycentrum (Begoniaceae) from southern Guangxi of China. PhytoKeys 182: 113–124. 10.3897/phytokeys.182.69074 34720624 PMC8516824

[B5] Forrest LL Hollingsworth PM (2003) A recircumscription of *Begonia* based on nuclear ribosomal sequences. Plant Systematics and Evolution 241(3–4): 193–211. 10.1007/s00606-002-0033-y

[B6] Gu CZ Peng CI Turland NJ (2007) Begoniaceae. In: Wu ZY Raven PH Hong DY (Eds) Flora of China (Vol.13). Science Press & Missouri Botanical Garden, Beijing & St. Louis, Missouri, 153–207.

[B7] Hall TA (1999) BioEdit: A user-friendly biological sequence alignment editor and analysis program for Windows 95/98/NT. Nucleic Acids Symposium Series 41: 95–98.

[B8] iBegonia (2021) The 2021 Report of *Begonia* Diversity in China [In Chinese]. https://mp.weixin.qq.com/s/qfap-qZVUhAJDnCrjTOCSg [accessed 10 October 2021]

[B9] IUCN (2022) Guidelines for Using the IUCN Red List Categories and Criteria. Version 15. Prepared by the Standards and Petitions Committee of the IUCN Species Survival Commission. https://www.iucnredlist.org/documents/RedListGuidelines.pdf [accessed 31 March 2022]

[B10] Liu Y Tseng YH Yang HA Hu AQ Xu WB Lin CW Kono Y Chang CC Peng CI Chung KF (2020) Six new species of *Begonia* from Guangxi, China. Botanical Studies (Taipei, Taiwan) 61(1): 21. 10.1186/s40529-020-00298-y PMC7393003 32734318

[B11] Moonlight PW Ardi WH Padilla LA Chung KF Fuller D Girmansyah D Hollands R Jara-Muñoz A Kiew R Leong W-C Liu Y Mahardika A Marasinghe LDK O’Connor M Peng C-I Pérez ÁJ Phutthai T Pullan M Rajbhandary S Reynel C Rubite RR Sang J Scherberich D Shui Y-M Tebbitt MC Thomas DC Wilson HP Zaini NH Hughes M (2018) Dividing and conquering the fastest-growing genus: Towards a natural sectional classification of the mega-diverse genus *Begonia* (Begoniaceae). Taxon 67(2): 267–323. 10.12705/672.3

[B12] Nylander JAA (2004) MrModeltest v2. Program distributed by the author. Evolutionary Biology Centre, Uppsala University. http://www.softpedia.com/get/Science-CAD/MrModeltest.shtml [accessed 28 April 2022]

[B13] Ronquist F Huelsenbeck JP (2003) MrBayes 3: Bayesian phylogenetic inference under mixed models. Bioinformatics 19(12): 1572–1574. 10.1093/bioinformatics/btg180 12912839

[B14] Shui YM Chen WH (2018) *Begonia* of China. Yunnan Science & Technology Press, Kunming, 285 pp.

[B15] Shui YM Huang SH (1999) Notes on the genus *Begonia* from Yunnan. Acta Botanica Yunnanica 21(1): 11–23.

[B16] Tian DK Xiao Y Tong Y Fu NF Liu QQ Li C (2018) Diversity and conservation of Chinese wild begonias. Plant Diversity 40(3): 75–90. 10.1016/j.pld.2018.06.002 30175289 PMC6114263

[B17] Tian DK Ge BJ Xiao Y Tian Q Li C (2021) *Begoniascorpiuroloba*, a new species in Begoniasect.Platycentrum (Begoniaceae) from southern Guangxi of China. Phytotaxa 479(2): 191–197. 10.11646/phytotaxa.479.2.5

[B18] Wu CY Ku TC (1995) New Taxa of the *Begonia* L. (Begoniaceae) from China. Acta Phytotaxonomica Sinica 33(3): 251–280.

[B19] Wu CY Ku TC (1997) New Taxa of the *Begonia* L. (Begoniaceae) from China (Cont.). Journal of Systematics and Evolution 35(1): 43–56.

